# Diagnostic value of transcranial ultrasonography for selecting subjects with large vessel occlusion: a systematic review

**DOI:** 10.1186/s13089-019-0143-6

**Published:** 2019-10-22

**Authors:** Daria Antipova, Leila Eadie, Ashish Stephen Macaden, Philip Wilson

**Affiliations:** 1 Centre for Rural Health, University of Aberdeen, Old Perth Road, Inverness, IV2 3JH UK; 2 Department of Stroke and Rehabilitation Medicine, Raigmore Hospital, NHS Highland, Inverness, IV2 3UJ UK

**Keywords:** Large vessel occlusion, Stroke, Acute cerebral ischaemia, Intracerebral haemorrhage, Transcranial ultrasonography, Neuroimaging

## Abstract

**Introduction:**

A number of pre-hospital clinical assessment tools have been developed to triage subjects with acute stroke due to large vessel occlusion (LVO) to a specialised endovascular centre, but their false negative rates remain high leading to inappropriate and costly emergency transfers. Transcranial ultrasonography may represent a valuable pre-hospital tool for selecting patients with LVO who could benefit from rapid transfer to a dedicated centre.

**Methods:**

Diagnostic accuracy of transcranial ultrasonography in acute stroke was subjected to systematic review. Medline, Embase, PubMed, Scopus, and The Cochrane Library were searched. Published articles reporting diagnostic accuracy of transcranial ultrasonography in comparison to a reference imaging method were selected. Studies reporting estimates of diagnostic accuracy were included in the meta-analysis.

**Results:**

Twenty-seven published articles were selected for the systematic review. Transcranial Doppler findings, such as absent or diminished blood flow signal in a major cerebral artery and asymmetry index ≥ 21% were shown to be suggestive of LVO. It demonstrated sensitivity ranging from 68 to 100% and specificity of 78–99% for detecting acute steno-occlusive lesions. Area under the receiver operating characteristics curve was 0.91. Transcranial ultrasonography can also detect haemorrhagic foci, however, its application is largely restricted by lesion location.

**Conclusions:**

Transcranial ultrasonography might potentially be used for the selection of subjects with acute LVO, to help streamline patient care and allow direct transfer to specialised endovascular centres. It can also assist in detecting haemorrhagic lesions in some cases, however, its applicability here is largely restricted. Additional research should optimize the scanning technique. Further work is required to demonstrate whether this diagnostic approach, possibly combined with clinical assessment, could be used at the pre-hospital stage to justify direct transfer to a regional thrombectomy centre in suitable cases.

## Introduction

### Rationale

Stroke is the second largest cause of death and one of the most common causes of complex disability in adults worldwide [1]. Mechanical thrombectomy (MT) preceded by intravenous thrombolysis provides optimal care for patients with acute ischaemic stroke due to large vessel occlusion (LVO) [2]. While thrombolysis is offered in many general hospitals, MT can only be performed in specialised endovascular centres with neurointerventional facilities. Earlier initiation of endovascular reperfusion is associated with greatly improved outcome [3, 4]. Therefore, patients with suspected LVO could benefit from direct transfer to a specialised endovascular centre.

A number of clinical assessment tools have been developed to triage subjects, but their false negative rates remain high which means that more than 20% of patients with LVO would be transferred to a centre with no dedicated facilities available [5]. Furthermore, false positive results can lead to unnecessary and costly emergency transfers. Hence, there is an urgent need to establish a reliable process to select patients with LVO, particularly in areas with long transport times.

Portable transcranial ultrasound (see Glossary for explanations of the different ultrasound types) has emerged as a potential method for rapidly assessing intracranial circulation and brain structures in the pre-hospital and early hospital phase [6]. The evidence base for the diagnostic accuracy of transcranial ultrasonography in acute stroke population appears to be extensive, with more than 150 publications. Up to date, it has not been demonstrated whether ultrasonography can be implemented as part of a triage system for patients with LVO to streamline care. A systematic analysis of the existing data in the field was therefore performed.

### Objectives

The following questions are addressed in the current review:

1. What is the diagnostic accuracy of transcranial ultrasonography in detecting occlusion and/or stenosis of cerebral vessels in acute ischaemic stroke population compared with other available diagnostic techniques?
2. Can transcranial ultrasonography be a useful tool to exclude intracranial haemorrhage as one of the contraindications for endovascular reperfusion compared with other available diagnostic techniques?
3. Can transcranial ultrasonography be a useful tool to detect midline shift suggestive of space-occupying stroke compared with other available diagnostic techniques?

## Methods

### Protocol and registration

The protocol is registered in PROSPERO, International prospective register of systematic reviews, and can be accessed at: https://www.crd.york.ac.uk/prospero/display_record.php?RecordID=75882.

### Eligibility criteria

Inclusion and exclusion criteria are listed in Table 1.

**Table 1:** Inclusion and exclusion criteria

Domain	Inclusion	Exclusion
Study type	Comparative observational studies	Case reports
	Prospective observational studies	Selected case series
	Cohort studies	Literature review
	Unselected case series	Conference proceedings
		Full text unavailable
Participants	Human	Non-human subjects
	Adults	Exclusively paediatric patients Mixed paediatric and adult populations (where paediatric and adult groups are not possible to identify separately)
	Patients with acute stroke—ischaemic (including patients with a transient ischaemic attack) or haemorrhagic	Patients with non-stroke conditions, such as sickle cell disease, arteriovenous malformation, traumatic brain injury, and cerebral tumour
	Patients with acute spontaneous subarachnoid haemorrhage	
Setting	Any	
Procedure	Transcranial ultrasonography (grey-scale/Doppler/colour-coded sonography) with/without contrast (microbubble) enhancement if the outcomes are reported separately	Transcranial ultrasonography (grey-scale/Doppler/colour-coded sonography) with contrast (microbubble) enhancement as sole ultrasound method, or if the outcomes are not reported separately
	A reference standard diagnostic tool, such as conventional imaging (CT, MRI), cerebral angiography (computed tomography angiography, magnetic resonance angiography, digital subtraction angiography)	No reference test employed
	Maximal time interval between the onset of symptoms and index and reference tests: 72 h	Unknown or more than 72 h’ time interval between the symptoms onset and index and/or reference tests
	Maximal time interval between the index and reference tests: 24 h	Unknown or more than 24 h’ time interval between the index and reference tests
Aims/outcomes	Detection of signs of acute cerebral ischaemia, acute intracranial haemorrhage, midline shift in space-occupying stroke as measured by both transcranial ultrasonography as index test and reference test	Detection of signs of vasospasm following subarachnoid haemorrhage

### Information sources

A systematic search of the literature was conducted during May–June 2017, using a database-specific search strategy for each of the following electronic databases: PubMed, Medline, Embase, Scopus and The Cochrane Library.

### Search

The search strategy included a combination of multiple iterations of MeSH and keyword terms relating to each component of the research question as outlined in Additional file 1.

The search was restricted to human studies, English language, and adult participants. There was no restriction for the year of publication.

### Study selection

**Fig. 1:**
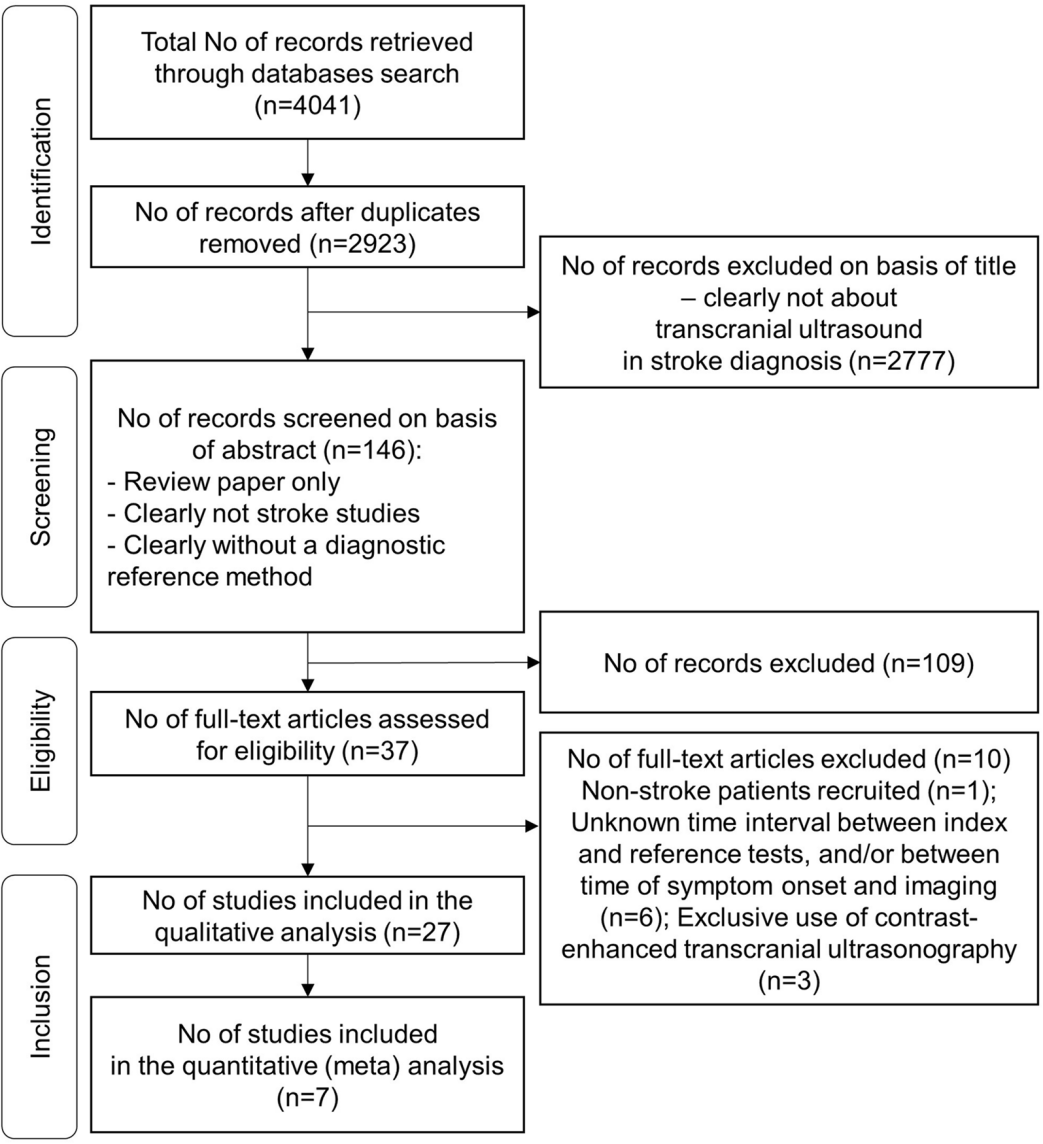
PRISMA flowchart. Outline of the study selection process using inclusion and exclusion criteria

The study selection process is illustrated in Fig. 1.

Eligible papers were tabulated and used in the qualitative synthesis. Studies which reported diagnostic accuracy values such as true positive (TP), false positive (FP), true negative (TN), false negative (FN), and sensitivity and specificity values were included in the quantitative analysis.

### Data collection and extraction

A dedicated data extraction form was developed and used to collect relevant information from the included studies. The inclusion of information fields in the data collection form was guided by the review questions. The following components were assessed:

- Study setting.
- Main characteristics of the patient population.
- Number of subjects recruited into the study and included in the study analysis.
- Ethical approval.
- Modality of transcranial ultrasonography being investigated, its technical considerations and qualifications of researchers performing transcranial ultrasound scanning.
- Reference test and technical considerations.
- Blindness of investigators performing and/or interpreting the tests’ results.
- Time interval from symptom onset to index and reference tests, time interval between index and reference tests.
- Whether results were presented in the form of diagnostic accuracy (sensitivity, specificity, TP, FP, TN, FN, predictive values), or a narrative.

Two of this review’s authors completed the data extraction form for each paper. If authors disagreed, a third author adjudicated. As our analysis concerned only published data, further data from investigators were not sought.

### Risk of bias assessment in individual studies

Included studies were assessed qualitatively for concerns regarding their applicability for each of the four domains: patient selection, index test, reference standard, and flow and timing, in accordance with the QUADAS-2 Tool quality assessment system [7]. The risk of bias and applicability concerns were identified using RevMan 5.3 software and presented as a summary table.

### Statistical analysis

The statistical analysis was performed in accordance with the Cochrane guidelines for diagnostic test accuracy reviews [8]. Two-by-two tables were constructed for the transcranial ultrasound results of diagnosis of acute steno-occlusion, intracranial haemorrhage, and detection of midline shift showing the binary test results cross-classified with the binary reference standard. The diagnostic values including the TP, TN, FP, and FN values were entered into RevMan 5.3. The sensitivity and specificity, as well as their 95% confidence intervals, were also calculated in RevMan 5.3. The data from each study were presented graphically by plotting sensitivities and specificities on a coupled forest plot and summary receiver operating characteristics (SROC) plot. Area under the curve for the SROC plot was calculated as a summary value for given sensitivities and specificities using IBM SPSS Statistics 25 package.

### Risk of bias assessment across studies

Provided that the number of studies reporting diagnostic accuracy values was ten or above, publication bias assessment with a funnel plot to illustrate the possibility of selective publication of small studies with positive results would be performed.

## Results

### Study selection

The results of the study selection process are illustrated in Fig. 1.

### Study characteristics

#### Included studies

27 studies in which participants were subjected to both transcranial ultrasonography and gold standard diagnostic imaging for diagnosing acute stroke were eligible for inclusion in the final analysis. In all studies, transcranial ultrasound was performed within 72 h of stroke symptom onset. Time interval between the index and reference tests did not exceed 24 h. Basic characteristics of included papers are presented in Table 2.

**Table 2:** Characteristics of included studies

Reference	Study setting	Condition of interest	Index test	Reference test	Number of patients included in the analysis	Time interval from symptoms onset to index test	Time interval between index test and reference imaging	Experience of the researcher performing transcranial ultrasonography	Type of transducer	Frequency of insonation
Akopov and Whitman [19]	In-hospital	AIS	TCD	MRA, CT	12	Only the results of TCD that was performed within 24 h after ictus were analysed	MRA was performed together with the first TCD examination for seven patients (included in the analysis). For all others, MRA was performed 24–72 h after the ictus and close to the second TCD (not included in the analysis)	Unclear—TCD was performed by the authors	Not specified	2 MHz
Bar et al. [23]	In-hospital	AIS	TCCS ± contrast enhancement	CTA	31	Within 3 h	20 min	Three skilled sonographers with at least 5 years of experience in ultrasound diagnostics	Sector	2–4 MHz
Boddu et al. [24]	Unclear—“in the laboratory”	AIS	TCD	MRA	128	Not specified—within 24 h in 33% of participants and within maximum 29 h in 67% of participants	From 30 to 300 min (median 60 min)	Credentialed neurosonologist	Not specified	2 MHz
Brunser et al. [25]	In-hospital	AIS	TCD (PMD)	CTA	100	Mean 468 min ± SD 343.2 min	Mean 77.8 min ± SD 88.5 min	Experienced sonographer certified by the American Society of Neuroimaging	Not specified	
Gerriets et al. [26]	In-hospital	AIS, MLS	TCCS	CT	40	Only results of TCCS examination that were performed 8 ± 3 and 16 ± 3 h from onset were analysed	6 h	Unclear—“three investigators”	Sector	2.5 MHz
Gerriets et al. [27]	In-hospital	AIS	TCCS ± contrast enhancement	CTA, MRA, DSA	58	Immediately on admission, within 6 h from symptom onset (mean 3.4 h)	Mean time difference = 0.8 h in 14 patients; 6.1 in 18 patients	Doctors with at least 1 year of experience in the field of colour-coded duplex sonography of the brain-supplying arteries	Sector	2–2.5 MHz
Goertler et al. [41]	Presumably in-hospital, department of neurology	AIS	TCCS	Contrast-enhanced TCCS	23	Within 5 h	Not specified but both tests performed within 5 h from symptom onset	A sonographer	Sector	2–2.5 MHz
Guan et al. [28]	In-hospital	AIS	TCD	CTA	128	The mean time from symptom onset to admission was 12.3 (10.1) hours The mean time from admission to TCD was 15.5 (SD 10.1) minutes	The mean time interval between both examinations was 89.7 (77.8) min 65% patients—less than 30 min difference between them; 25%, 31–90 min; 15%, more than 90 min but less than 180 min	Experienced sonographer	Not specified	2 MHz
Kadimi et al. [43]	Not specified—presumably in-hospital	AIS	TCD	CT	4	Within 6 h	Not specified—both CT and TCD were performed within 6 h of the onset of symptoms	Not specified	Sector	2 MHz
Kenton et al. [29]	In-hospital	AIS	TCCS	MRA	30	Ranged from 4 to 24 h, mean 15.4 h	Within 4 h, range 15 min to 4 h; median, 2 h	Not specified	Curved phase array	2.25 MHz
Kern et al. [30]	In-hospital, stroke unit	ICH	Native transcranial b-mode ultrasound, UPI with contrast enhancement	CT	12	Unclear—on day 1 as soon as possible after admission	4.1 ± 2.5 h on day 1 (CT first)	Sonographers	Sector	2–4 MHz
Kukulska-Pawluczuk et al. [44]	In-hospital	ICH, MLS	TCCS	CT	39	The time between initial symptoms of focal neurologic deficit and hospital admission ranged from 1.5 to 48 h with a median of 5.9 h. Index test was performed not later than 12 h after initial CT which was done directly upon admission	Not more than 12 h	Unknown	Sector	2.5 MHz
Leanyvari et al. [45]	Unclear	AIS	TCD	CT	12	Within 12.5 ± 8 h after stroke onset or not more than 24.5 h	TCD measurements were made before or no more than 4 h after CT	Not specified	Not specified	2 MHz
Matsumoto et al. [31]	In-hospital	ICH	TCCS	CT	20	Within 21 h (within 12 h of the CT study which was performed 4.6 ± 4.4 h from symptom onset)	Within 12 h (mean 3.9 ± 4.1 h)	Not specified	Sector	2.5 MHz
Nasr et al. [32]	Outpatient, TIA clinic	AIS	TCCS	MRI-3D-TOF angiography	116	Unclear but presumably within 24 h from the onset	4 h	Unclear	Not specified	
Ovesen [42]	In-hospital	ICH	Transcranial b-mode ultrasound	CT, CTA	25	Within 4.5 h	Mean 61.1 min (SD 26.6)	Unclear (“a single observer”)	Sector	1.7–3.1 MHz
Panerai et al. [20]	In-hospital	AIS	TCD	MRI-DWI	11 plus 9 healthy controls	Within 48 h	Median time interval 2 h (range 0.5–8 h)	Unclear—performed “in a dedicated cardiovascular research laboratory”	Not specified	2 MHz
Rathakrishnan et al. [33]	In-hospital	AIS	TCD	CTA	15	Not specified	Within 24 h	Not specified, a stroke neurologist credentialed in cerebrovascular ultrasound interpreted the TCD findings	Not specified	2 MHz
Seidel et al. [34]	In-hospital	AIS, HT	TCCS	CT	32	For the purpose of the current review only TCCS findings which were obtained < 12 and 24 ± 4 h from symptoms onset were analysed	Unclear but before TCCS	Sonographer	Sector	2–4 MHz
Seidel et al. [35]	In-hospital	AIS, HT	TCCS	CT, MRI in individual cases	55	Within 32 h (mean time 10.6 h (SD, 7.2; median, 8.5; interquartile range, 6.5 h after stroke symptom onset)	Within mean time of 14 h (CT was performed immediately after stroke symptom onset with mean 3.3 h; SD, 3.0; median, 2.0; interquartile range, 3.75)	The ultrasound investigator	Sector	2 MHz
Stolz et al. [36]	In-hospital	AIS, ICH, MLS	TCCS	CT	61	Unclear, presumably within 24 h	3–12 h	Four sonographers with sufficient experience with the method	Not specified	2–2.5 MHz
Tang et al. [37]	In-hospital, stroke unit	ICH, MLS	TCCS	CT	51	Unclear but presumably within 24 h because time from symptom onset to reference imaging was 4.1 ± 3.7 h	Not more than 12 h, average interval was 5.9 ± 4.0 h	Well-trained and experienced sonographers	Sector	2 MHz
Tsivgoulis et al. [38]	Emergency department	AIS	TCD	CTA	132	Within 24 h	Range 10–130 min (median 35 min)	Experienced sonographers	Not specified	2 MHz
Tsivgoulis et al. [39]	Emergency room	AIS	TCD (PMD)	CTA, MRA, DSA	213	Within 24 h	Presumed 24 h, angiography was performed within 48 h from ictus	Stroke neurologists with specialised training and credentials in cerebrovascular ultrasound	Not specified	2 MHz
Viola et al. [21]	In-hospital	AIS	TCD (3D)	MRA, CT	47 plus 67 healthy controls	Within 3–24 h	Unclear, presumably not more than 24 h—both tests were performed 3–24 h from onset	Not specified	Not specified	2 MHz
Wada et al. [40]	Not specified	AIS	TCCS	DSA	40	Within 24 h	Not specified—“immediately before cerebral angiography”	Unclear	Not specified	2–3 MHz, 3700 Hz pulse repetition frequency, low-pass filter was 50 Hz
Zubkov et al. [46]	In-hospital	AIS	TCD	CTA	31	Approximately 30 h after symptom onset	Unclear, presumably within 24 h	Experienced ultrasonographers	Not specified	2 MHz

For the purpose of the current review, terminology used to describe an ultrasound probe has been standardised and phased array, sector and pulsed wave Doppler are referred to as the same type of probe

*3D* three-dimensional, *AIS* acute ischaemic stroke, *CT* computed tomography, *CTA* computed tomography angiography, *DSA* digital subtraction angiography, *ICH* intracranial haemorrhage, *HT* haemorrhagic transformation, *MRA* magnetic resonance angiography, *MRI-3D-TOF* magnetic resonance three-dimensional time-of-flight imaging, *MRI-DWI* diffusion weighted magnetic resonance imaging; *PMD* power motion-mode, *SD* standard deviation, *TCCS* transcranial colour-coded duplex sonography, *TCD* transcranial Doppler, *UPI* ultrasound perfusion imaging

All papers described prospective observational studies. In total, 1683 participants (patients *n* = 1566, controls *n* = 117) were analysed across the 27 study populations. Individual studies varied considerably in sample size, from 4 to 213 subjects, and consisted of participants recruited upon presentation to the hospital or outpatient departments. There were no pre-hospital studies identified that would meet the inclusion criteria for the current systematic review.

The search did not retrieve any eligible studies aimed to assess accuracy of transcranial ultrasonography for detecting signs of subarachnoid haemorrhage (SAH).

Seven papers presented study results reporting quantitative values of the diagnostic accuracy of the transcranial ultrasonography (TP, TN, FP, FN, sensitivity, and specificity), whereas 20 discussed outcomes qualitatively or did not fully report diagnostic accuracy values.

On the basis of their full-text articles, ten studies were not included in the final analysis [9–18]. Principal reasons were unknown or unclear time interval between symptom onset and reference/index imaging and/or between reference and index imaging [9, 10, 12, 14–18], the use of contrast-enhanced transcranial sonography either exclusively or in combination with non-contrast ultrasound without reporting outcomes separately for each group [9, 11, 13], or a non-stroke population [12].

### Risk of bias assessment in individual studies

A summary of bias and applicability concerns is presented in Table 3.

**Table 3:** Risk of bias and applicability concerns summary

	Risk of bias				Applicability concerns		
	Patient selection	Index test	Reference standard	Flow and timing	Patient selection	Index test	Reference standard
Akopov and Whitman [19]	High	Low	Unclear	Low	Low	Low	Low
Bar et al. [23]^a^	Low	Low	Low	High	Low	Low	Low
Boddu et al. [24]	Low	Low	Low	Low	Low	Low	Low
Brunser et al. [25]^a^	Low	Low	Low	Unclear	Low	Low	Low
Gerriets et al. [26]	Low	Low	Low	Low	Low	Low	Low
Gerriets et al. [27]	Low	Low	Low	High	Low	Low	Low
Goertler et al. [41]	Unclear	High	High	Unclear	High	Low	Low
Guan et al. [28]^a^	Unclear	Low	Low	Low	Unclear	Low	Low
Kadimi et al. [43]	Unclear	Unclear	Unclear	Low	Low	Low	Low
Kenton et al. [29]	Unclear	Low	Low	High	Low	Low	Low
Kern et al. [30]	Low	Low	Low	Low	Low	Low	Low
Kukulska-Pawluczuk et al. [44]	Unclear	Unclear	Low	High	Low	Low	Low
Leanyvari et al. [45]	High	Low	High	Low	Low	Low	High
Matsumoto et al. [31]	Low	Low	Low	Unclear	Low	Low	Low
Nasr et al. [32]	Low	Low	Low	High	Low	Low	Low
Ovesen et al. [42]	Low	High	Low	High	Low	Low	Low
Panerai et al. [20]	High	High	High	Low	High	Low	High
Rathakrishnan et al. [33]^a^	High	Low	Low	Unclear	Low	Low	Low
Seidel et al. [34]	High	Low	Unclear	Low	Low	Low	Low
Seidel et al. [35]	Unclear	Low	Unclear	Low	Low	Low	Low
Stolz et al. [36]	Unclear	Low	Unclear	Low	Low	Low	Low
Tang et al. [37]	Low	Low	Unclear	High	Low	Low	Low
Tsivgoulis et al. [38]^a^	Low	Low	Low	Low	Low	Low	Low
Tsivgoulis et al. [39]^a^	Unclear	Low	Low	Low	Low	Low	Low
Viola et al. [21]	High	Unclear	High	High	Low	Low	High
Wada et al. [40]^a^	Low	Low	Low	Low	Low	Low	Low
Zubkov et al. [46]	Unclear	High	Unclear	High	Low	Unclear	Low

Review author’s judgement about each domain for each included study

Studies marked with an “^a^” were included in the quantitative analysis

Case–control methodologies were employed in three included studies [19–21] in which groups of patients with and without the target condition were identified before the index test was performed.

The papers were also evaluated in regard to the sample size and divided into three groups [22]: low sample size (< 100 participants), intermediate (100–300 participants), and high (> 300 participants). Only six studies had intermediate sample size, whereas in the remaining 21 it was low.

Ethical approval was not reported in 44% (*n* = 12) of the included papers.

A list of transcranial ultrasonography modalities used in individual studies as an index test is provided in Table 2. All included studies were assessed against the adequate blinding criterion. In 19, the results of the index test were interpreted without knowledge of the results of the reference standard [19, 23–40]. Two studies [41, 42] were lacking blindness, and in 6 papers it was not explicitly mentioned whether or not the investigator was blinded to the results of the reference standard [20, 21, 43–46].

Studies in which transcranial ultrasound scanning and reference standards were performed on the same patient group and the whole sample should have received verification of the diagnosis by the reference standard were included. The control methods are listed in Table 2.

Although all studies that did not employ a reference imaging modality were excluded, there was inconsistency in application and interpretation of the various reference standard tests. Some of the studies used several imaging modalities as a reference test, and for most articles, it was either computed tomography angiography (CTA), magnetic resonance angiography (MRA), or digital subtraction angiography (DSA) [21, 25, 27, 39]. For example, in the paper by Gerriets et al. [27], the choice of reference test (CTA, MRA, or DSA) was left up to the treating centre.

### Diagnostic accuracy of transcranial ultrasonography in acute stroke

The diagnostic accuracy of transcranial ultrasonography in detecting signs of acute steno-occlusive lesions, intracranial haemorrhage and/or midline shift was a primary outcome in all studies. All included studies that specified the type of a probe and frequency of transcranial ultrasonography performed scanning with a sector probe at low frequency (2–4 MHz) (Table 2). Kenton et al. [29] used a curved phase array probe for their study, however, this did not seem to provide any additional and/or contradictory information to other reviewed studies.

The majority of included papers (22/27) aimed to evaluate diagnostic accuracy of transcranial Doppler sonography (TCD) in detecting signs of acute occlusion and/or stenosis in acute ischaemic stroke population (Table 2). However, there was marked diversity in diagnostic protocol and criteria for steno-occlusive lesions (Additional files 2 and 3).

Seventeen studies assessed diagnostic value of TCD in identifying occlusion of the cerebral arteries [19, 21, 23, 25, 27–29, 32, 33, 35, 38–41, 43, 45, 46]. Diagnosis of the middle cerebral artery (MCA) mainstem occlusion can be considered when the flow was absent or minimal, blunted, or damped throughout the MCA while the flow in the distal internal carotid artery, anterior and posterior cerebral arteries was diverted [23, 27, 35, 38, 40]. Similarly, anterior cerebral artery occlusion and occlusion in the posterior circulation (vertebral, basilar and posterior cerebral arteries) were defined as the absence of the flow or the presence of minimal, blunted, or dampened flow signals throughout these vessels [38, 39].

The interhemispheric asymmetry index has been used to describe the difference in the blood flow velocity between symptomatic and asymptomatic hemispheres [47]. Asymmetry index ≥ 21% was shown to correlate accurately with clinical presentation and to detect major branch or multiple branch occlusion [21, 23, 29, 35, 41]. In patients with an asymmetry index of < 21%, conventional imaging (CT, MRI) is likely to offer normal appearance or show only very minor changes suggestive of acute brain ischemia [21, 28, 29]. Kadimi et al. [43] reported a hyperechoic appearance of MCA with no Doppler signal or waveform suggestive of a large occlusion, similar to hyperdense MCA on CT. Occlusion of one or two terminal branches of the MCA presenting as lacunar stroke has been reported to be “insufficient” to cause changes detectable with TCD [47].

**Fig. 2:**
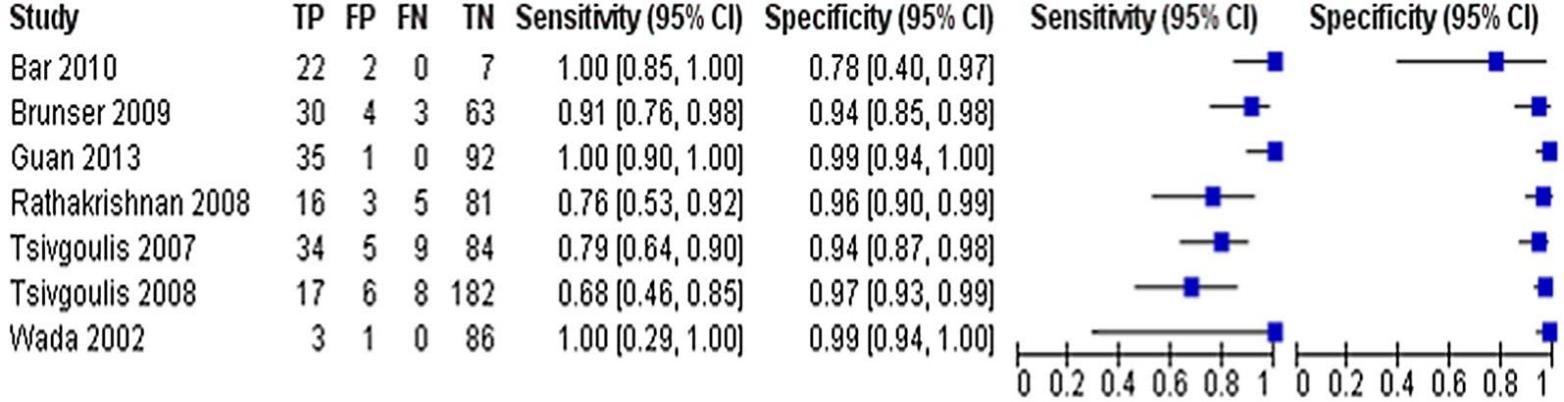
Forest plot. Estimated diagnostic accuracy of transcranial ultrasonography in detecting steno-occlusive lesions in acute stroke population

**Fig. 3:**
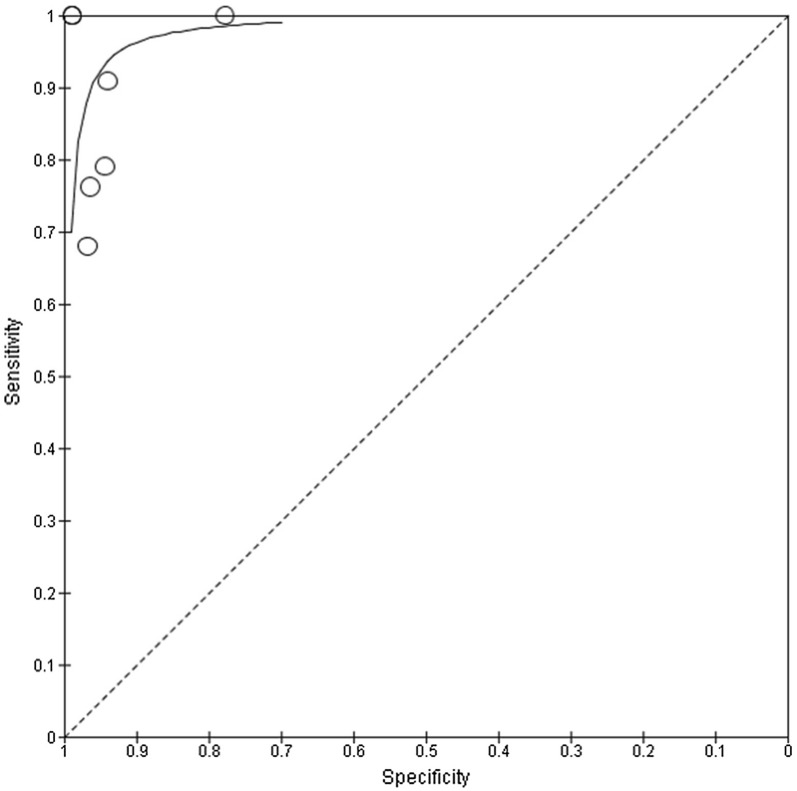
Summary receiver operating characteristic plot. Each circle represents the sensitivity and specificity estimate from one study

Diagnostic accuracy values for the detection of cerebral steno-occlusive lesions in comparison to angiography as the reference test (CTA [23, 25, 28, 33, 38], CTA/MRA/DSA [39], DSA [40]) are presented in the forest plot (Fig. 2) and SROC plot (Fig. 3).

In general, there was a good correlation between TCD and reference standard findings in the acute ischaemic stroke population, with the sensitivity ranging from 68 to 100% and the specificity 78–99%. The area under the SROC curve was 0.91. However, TCD appeared to be more sensitive in detecting MCA steno-occlusive lesions when compared to assessment of the posterior cerebral circulation but was less specific (Additional file 4) [33, 38],

Transcranial ultrasonography can also provide evidence of intracranial haemorrhage in some cases. Acute haemorrhagic foci are usually seen on ultrasound images as homogenous hyperechogenic structures that are well distinguished from surrounding tissues. Transcranial ultrasound has been shown to detect 52.9% of haemorrhagic foci in deep cerebral structures, such as basal ganglia, whereas cortico-subcortical lobar lesions were missed in about 67% of cases, mainly in the temporal and frontal lobes [30, 34, 44], There was a good correlation between the volumes of haemorrhagic foci measured by transcranial ultrasound and CT [31, 42, 44]. It revealed haemorrhagic foci with volumes as small as 0.47 mm^3^ and as large as 234 mm^3^ [44].

Although transcranial ultrasonography has been shown to identify haemorrhage in supratentorial and infratentorial locations, and intraventricular haemorrhages [42], echogenicity of haemorrhage appears to be similar to that of the cerebellar tentorium and calcifications within choroid plexuses. Cerebral tumours, vascular malformations and cerebral microangiopathies may also appear as hyperechogenic areas mimicking haemorrhagic lesions [44, 48].

Transcranial ultrasound has been demonstrated to be a useful diagnostic tool in detecting midline shift in space-occupying haemorrhagic or ischaemic stroke lesions [42]. There was a good consistency between the studies in the technique used to measure midline shift as a deviation from the presumed midline ipsilaterally and contralaterally to the focus as a distance between the source of the ultrasound beam and the centre of the third ventricle.

There was an excellent correlation between transcranial ultrasonography and CT measurements of the midline shift with a coefficient of about 0.9 [26, 36, 37, 44]. Midline shift has been reported to be the most sensitive indicator of intracranial haemorrhage with a significant correlation between haemorrhagic focus volume and midline shift [37, 44].

### Ultrasound window availability

One of the technical limitations of transcranial ultrasonography is the inability to visualise brain structures of interest in patients who have an inadequate temporal bone acoustic window.

The average incidence of temporal acoustic window failure in the included studies was 19.7%, which led to false negative results in a number of cases [27]. Notably, in the study by Matsumoto et al. [31], the majority of patients were Asian, and there was a higher reported rate of temporal window failure of 56.3% of cases (27/48).

### Risk of bias assessment across studies

Since only seven papers presented study results reporting quantitative values of the diagnostic accuracy of the test, publication bias assessment illustrating the possibility of selective publication of small studies with positive results was not performed.

## Discussion

There is a substantial number of published diagnostic accuracy evaluations of transcranial ultrasonography in acute stroke population. 27 English-language studies that compared ultrasonography with other conventional imaging tools within 72 h of stroke symptom onset and with not more than 24 h’ time difference between the index and reference tests were identified.

Different types of transcranial ultrasonography were used in the studies selected for the final analysis. TCD appeared to be the most commonly employed techniques, which could be explained by the fact that more than 80% of the papers aimed to evaluate cerebral blood flow and to detect signs of acute occlusion and/or stenosis.

Seventeen studies included in the current review evaluated diagnostic value of TCD in identifying patients with acute vessel occlusion. Thus, absent or diminished Doppler signal in the MCA mainstem is suggestive of its occlusion [38]. Asymmetry index equal or higher than 21% is considered indicative of a large branch or multiple branch occlusion. It is less likely to be of diagnostic value in lacunar stroke cases as blood flow abnormalities caused by MCA terminal branch occlusion might not be detected with TCD [21, 29, 47]. A finding of hyperechoic MCA signal is comparable to a hyperdense MCA on CT and suggestive of a large vessel occlusion [43].

The sensitivity and specificity for detecting steno-occlusive lesions in cerebral arteries were 68–100% and 78–99%, respectively, with the highest specificity for MCA lesions. These findings could potentially be used for the selection of subjects with LVO for direct transfer to an endovascular centre. Its application for detection of distal MCA stenosis [46] and steno-occlusive lesions in the posterior cerebral circulation is, however, limited. This might be explained by a suboptimal angle of insonation which may lead to ambiguous findings [23, 24, 29, 33, 38]. This limitation can be potentially overcome by obtaining angle-corrected flow velocities [24].

There was a significant variation in diagnostic criteria used to detect acute steno-occlusive lesions. The lack of any approved diagnostic protocol for assessing cerebral artery occlusion and stenosis represents a big challenge as the results of studies are not absolutely compatible. There are a few recognised limitations of TCD, such as an inability to visualise anatomical landmarks, and as a consequence, a risk of inaccurate classification of specific blood vessels. Identification of blood vessels usually proceeds on the basis of indirect parameters, in particular: depth of insonation, flow direction and transducer orientation and position.

Differentiation between haemorrhagic and ischemic stroke is an important step in determining patient pathways. Transcranial ultrasonography can be used in some cases to detect intraparenchymal bleeds located in the brain structures that can be easily visualised with ultrasonographic examination, such as the basal ganglia. However, imaging of cortical regions, specifically frontal and parietal lobes, is largely restricted by the low spatial resolution of transcranial ultrasonography in these areas. It has also been reported that trace amounts of blood in the ventricular system may be missed, specifically in the occipital horn of the lateral ventricle [17]. Furthermore, some pathological lesions within the brain structures, such as calcifications, tumours, and vascular malformation can mimic the appearance of intracranial haemorrhage, and therefore may lead to false positive ultrasonographic findings [44, 48]. Midline shift might be used as an indirect parameter suggestive of increased intracranial pressure due to space-occupying stroke lesion [26, 42].

Our search also did not identify any eligible papers assessing diagnostic accuracy of transcranial ultrasonography in detecting signs of spontaneous SAH that have been described elsewhere as a hyperechogenic signal in the basal cisterns [49]. Studies evaluating diagnostic accuracy of transcranial ultrasonography for the detection of vasospasm after SAH were excluded from the current review.

Transcranial ultrasound is a simple, non-invasive and affordable diagnostic tool that can be repeated as many times as required at the patient’s bedside and in space-restricted environments, such as ambulances [31, 44, 50, 51]. It takes on average 4.3–13.6 min for the complete examination of cerebral vessels [27, 41]. It can provide useful information complementary to clinical assessment, serving as a “stethoscope for the brain” [28]. A fast-track protocol for contrast-enhanced duplex ultrasound assessment in the Emergency Department has been suggested by Connolly et al. [52]. They demonstrated that the mean time to complete the ultrasound examination reduced on average by 1 min 46 s if performed by more experienced sonographers. As demonstrated previously [50, 51, 53], transcranial ultrasound images can be transferred for expert interpretation from remotely supported ambulances in remote and rural areas.

There are, however, a few limitations that restrict its use from being more widespread: namely the operator-dependent nature of interpretation, and the possibility of patients having inadequate temporal acoustic windows. Temporal bone acoustic window failure has been reported in 8–29% of the general population, and is more commonly seen in women over the age of 50 years and the Asian population [54, 55]. Contrast-enhanced transcranial ultrasonography may be considered as a possible solution, since it has been shown to provide a better visualisation compared with non-enhanced examination [41]. Further research is required for technique optimization of transcranial ultrasonography with the potential application of lower frequency, which may provide greater penetration through the skull into the brain.

Early definitive imaging in patients suspected of having suffered an acute stroke is desirable. However, rapid performance of a CT or angiography is unrealistic in many remote regions of the world requiring expensive and sometimes hazardous transfers to hospital. The gathered evidence suggests that transcranial ultrasound assessment alone currently does not allow an accurate diagnosis particularly in haemorrhagic stroke cases which is the main requirement for this technique to be used in the reliable pre-hospital triage process. However, its diagnostic accuracy might be improved by adding clinical assessment [5] to justify transfer to hospital associated with high costs and risks.

### Limitations and bias

Given the highly specific nature of the literature search, almost all of the relevant literature available is believed to have been retrieved. However, papers in languages other than English were not included. Some papers reporting negative results in their evaluation of diagnostic accuracy of transcranial ultrasonography in acute stroke were not included, since they did not meet our inclusion criteria [18, 56]. Thus, Kamal et al. [18] studied cerebral artery stenosis in stroke patients from a South Asian population, and concluded that TCD has poor sensitivity for detection of arterial stenosis when compared to MRA. This study was not included in the qualitative analysis because of the unclear time interval between stroke onset and examinations and between index and reference tests. Suwanwela et al. [56] reported that TCD should not be used as a screening tool for identifying stenotic lesions in MCA territory stroke since a normal TCD examination does not exclude stenosis of the distal MCA branches. This paper was excluded from the final analysis as the time interval between TCD and CTA could exceed 24 h. However, none of the above studies reported findings that would contradict the results of the other reviewed papers.

Three studies that employed case–control methodology to compare transcranial ultrasound findings in patients with acute stroke and a healthy age-matched population were identified. Case–control studies have been shown to report two- or threefold higher estimates of diagnostic accuracy when compared to single series studies (spectrum bias) [57].

Most of the studies were relatively small, with the number of recruited patients being less than 100, and all were conducted in in-hospital settings. This might indicate potential selection bias. The present systematic review was not restricted to studies recruiting consecutive participants, however, a protocol which allows researchers to select individuals for inclusion in the study may also have selection bias.

One source of potential biases in the methodology relating to the reference test involved lack of blinding of investigators performing or interpreting results. This might affect the interpretation of the results potentially leading to either over- or underestimated performance of the reference test.

A time interval of not more than 24 h between the index and reference imaging to ensure minimal discrepancy between studies due to evolution of changes as a result of hypoxia or thrombus propagation, dissolution, or reocclusion [28, 38] was one of the inclusion criteria for the present systematic review. Therefore, this source of bias was not identified in any of the papers. However, the timing of the diagnostic tests was often not specifically reported as within 24 h but, for instance, “on admission”.

Some potential biases were identified indicated by inconsistent reporting of important methodological details, such as level of expertise of the person performing the investigation with transcranial ultrasonography and blindness of investigators performing and/or interpreting reference and index tests. In the majority of the studies, ultrasonography investigation was performed by a specialist, reducing the risk of potential diagnostic bias in these cases. It is important to ensure that operators performing or interpreting transcranial ultrasound scans have specialist knowledge and adequate experience in the field if their assessment results are to be used to guide management of acute stroke patients. Inexperienced transcranial ultrasound users might potentially misinterpret findings leading to higher rates of false results and consequent wrong management decisions.

It appears that in most studies the index and reference tests were interpreted independently, without knowledge of the other’s findings. About 37% (10/27) of reviewed studies included fewer than 80% of the participants in the analysis of outcomes and results which may introduce bias in the studies if there is some systematic difference between the excluded participants and those included in the final analysis.

In some studies, several imaging modalities were used as reference tests [21, 25, 27, 39]. The choice of imaging modality in acute stroke is guided by different factors, including suspected type of stroke, the time from symptom onset, contraindications for specific diagnostic tool, etc. However, such variability across the study population might lead to misinterpretation of the diagnostic accuracy of the index test.

Currently, a sector (cardiac) probe is most commonly used for the purposes of transcranial ultrasonography, since it has a small footprint and allows scanning at low frequency (2–4 MHz). However, it does not allow a continuous satisfactory visualisation of brain structures within the acoustic window during the scanning due to its poor conformity with temporal bone anatomy. Future work is needed to develop a probe that would be anatomically suitable specifically for transcranial scanning and allows imaging at angles that would allow better coverage of the brain.

## Conclusions

This systematic review demonstrates that transcranial ultrasonography might potentially be used for the selection of subjects with acute LVO, to help streamline patient care and allow direct transfer to specialised endovascular centres. It can also assist in detecting haemorrhagic lesions in some cases, however, its applicability here is largely restricted. Further work is needed to establish a formal diagnostic protocol and criteria for detecting signs of acute stroke, to ensure consistency in reporting results. Additional research should optimize the scanning technique at lower frequency with potential for construction of a dedicated probe that would be compatible with anatomical shape of the temporal bone windows. Future work is also required to demonstrate whether this diagnostic approach, possibly combined with clinical assessment, could be used at the pre-hospital stage to justify direct transfer to a regional thrombectomy centre in suitable cases.

## Supplementary information

**Additional file 1.** Search strategy.

**Additional file 2.** Criteria for the diagnosis of arterial stenosis in individual studies.

**Additional file 3.** Criteria for the diagnosis of arterial occlusion in individual studies.

**Additional file 4.** The accuracy parameters of transcranial ultrasonography for detecting cerebral arterial stenosis or occlusion in individual studies.

## Data Availability

Not applicable.

## Abbreviations

CT: computed tomography
CTA: computed tomography angiography
DSA: digital subtraction angiography
FN: false negative value
FP: false positive value
LVO: large vessel occlusion
MCA: middle cerebral artery
MRA: magnetic resonance angiography
MRI: magnetic resonance imaging
MT: mechanical thrombectomy
SAH: subarachnoid haemorrhage
SROC: summary receiver operating characteristics
TCD: transcranial Doppler sonography
TN: true negative value
TP: true positive value

## Glossary

Transcranial brightness mode (b-mode) sonography: Application of low-frequency ultrasound wave through a thin skull bone to enable a grey-scale visualisation of cerebral structures [58].
Transcranial Doppler sonography (TCD): A pulsed Doppler system with low transmitter frequency for recording blood flow velocities from basal cerebral arteries on the basis of indirect parameters: the depth of the sample volume, the position of the transducer, and the flow direction [59].
Transcranial colour-coded duplex sonography (TCCS): A method of ultrasonography that enables the visualisation of the basal cerebral arteries by colour coding of blood flow velocity; a combination of b-mode and Doppler ultrasonography [58].
