# Supplementary material for: Diagnostic value of transcranial ultrasonography for selecting subjects with large vessel occlusion: a systematic review
Source: Ultrasound J. 2019 Oct 22;11:29. doi: 10.1186/s13089-019-0143-6 (PMC6805840; doi:10.1186/s13089-019-0143-6)
Supplement: Supplementary file 1 — Additional file 1. Search strategy. [file 13089_2019_143_MOESM1_ESM.docx]

Additional file 1. Search strategy

| **Database** | **Search** |
| --- | --- |
| 1. EMBASE | ((exp brain hemorrhage or exp cerebrovascular accident or exp subarachnoid haemorrhage or exp brain hematoma or (brain h?emorrhag$ or intracranial h?emorrhag$).af. or cerebral h?emorrhag$.af. or (subarachnoid h?emorrhag$ or SAH).af. or h?ematoma.af.) or (exp brain infarction or exp cerebrovascular accident or exp brain ischemia or (acute isch?emic stroke or acute cerebral isch?emia).af. or exp cerebral artery disease) or (exp brain edema or (brain ?edema or cerebral ?edema).af. or midline shift.af.)) and ((exp transcranial Doppler ultrasonography or (transcranial colo?r-coded sonography or TCCS).af. or (transcranial ultrasound or transcranial ultrasonography or transcranial sonography).af.) and  (exp computer assisted tomography or (computed tomography or CT).af. or exp neuroimaging or exp functional neuroimaging or exp nuclear magnetic resonance imaging or (magnetic resonance imaging or MRI).af. or exp brain angiography or (computed tomography angiography or CT angiography).af. or (magnetic resonance angiography or MRA).af.)) limit 44 to (human and english language) |
| 1. MEDLINE | ((exp Intracranial Hemorrhages/ or exp Stroke or exp Cerebral Hemorrhage or exp Subarachnoid Hemorrhage or exp Hematoma or intracranial h?emorrhag*.af. or cerebral h?emorrhag*.af. or (subarachnoid h?emorrhag* or SAH).af. or h?ematoma.af.) or (exp Brain Infarction or exp Stroke, Lacunar or exp Brain Ischemia or exp Cerebral Infarction or exp Infarction, Middle Cerebral Artery or (acute isch?emic stroke or acute cerebral isch?emia).af.) or (exp Brain Edema or (brain ?edema or cerebral ?edema).af. or midline shift.af.)) and ((exp Ultrasonography, Doppler, Transcranial or (transcranial colo?r-coded sonography or TCCS).af. or (transcranial ultrasound or transcranial ultrasonography or transcranial sonography).af.) and (exp Tomography, X-Ray Computed or (computed tomography or CT).af. or exp Neuroimaging or exp Magnetic Resonance Imaging or (magnetic resonance imaging or MRI).af. or exp Cerebral Angiography or (computed tomography angiography or CT angiography).af. or (magnetic resonance angiography or MRA).af.)) limit 47 to (english language and humans) |
| 1. Pubmed | ((((((intracranial hemorrhages[MeSH Terms]) OR stroke[MeSH Terms]) OR subarachnoid hemorrhage[MeSH Terms]) OR cerebral hemorrhage[MeSH Terms]))))) OR ((((brain infarction[MeSH Terms]) OR stroke[MeSH Terms]) OR brain ischemia[MeSH Terms]) OR cerebral infarction[MeSH Terms]) OR infarction, middle cerebral artery[MeSH Terms])))) OR (brain edema[MeSH Terms])) AND (((((transcranial Doppler ultrasonography[MeSH Terms]) OR transcranial color-coded sonography[MeSH Terms]) OR transcranial ultrasound[MeSH Terms]) OR transcranial ultrasonography[MeSH Terms]) OR transcranial sonography[MeSH Terms]))))) AND (((((tomography, x-ray computed[MeSH Terms]) OR neuroimaging[MeSH Terms]) OR magnetic resonance imaging[MeSH Terms]) OR angiography[MeSH Terms]) OR computed tomography angiography[MeSH Terms]) OR magnetic resonance angiography[MeSH Terms])))))) Filters: Clinical Study; Clinical Trial; Comparative Study; Evaluation Studies; Meta-Analysis; Multicenter Study; Observational Study; Review; Humans; English; Adult: 19+ years |
| 1. SCOPUS | ( ( ( TITLE-ABS-KEY ( "tomography x-ray computed" OR ct OR "computed tomography" OR neuroimaging OR "magnetic resonance imaging" OR mri OR angiography OR "cerebral angiography" OR "computed tomography angiography" OR "CT angiography" OR "magnetic resonance angiography" OR mra ) ) ) AND ( TITLE-ABS-KEY ( "ultraso* Doppler transcranial" OR "transcranial Doppler ultraso*" OR tcd OR "transcranial colo?r-coded sonography" OR tccs OR "transcranial ultrasound" OR "transcranial ultrasonography" OR "transcranial sonography" ) ) ) AND ( ( TITLE-ABS-KEY ( "intracranial h?emorrhag*" OR stroke OR "subarachnoid h?emorrhag*" OR "cerebral h?emorrhag*" OR "brain h?emorrhag*" OR "cerebrovascular accident" OR h?ematom* ) ) OR ( TITLE-ABS-KEY ( "brain infarction" OR "cerebral infarction" OR "stroke" OR "cerebrovascular accident" OR "acute isch?emic stroke" OR "acute cerebral isch?emia" OR "brain isch?emia" OR "cerebral infarction" OR "middle cerebral artery infarction" ) ) OR ( TITLE-ABS-KEY ( "brain ?edema" OR "cerebral ?edema" OR "midline shift" ) ) ) AND ( LIMIT-TO ( DOCTYPE , "ar" ) OR LIMIT-TO ( DOCTYPE , "re" ) OR LIMIT-TO ( DOCTYPE , "ip" ) ) AND ( LIMIT-TO ( LANGUAGE , "English" ) ) AND ( LIMIT-TO ( SRCTYPE , "j" ) ) |
| 1. The Cochrane Library | ((MeSH descriptor: [Intracranial Hemorrhages] explode all trees or MeSH descriptor: [Stroke] explode all trees or MeSH descriptor: [Subarachnoid Hemorrhage] explode all trees or MeSH descriptor: [Cerebral Hemorrhage] explode all trees or MeSH descriptor: [Hematoma] explode all trees or "intracranial h?emorrhag*":ti,ab,kw or "subarachnoid h?emorrhag*":ti,ab,kw or "brain h?emorrhag*":ti,ab,kw or "cerebrovascular accident":ti,ab,kw or "h?ematom*":ti,ab,kw) or (MeSH descriptor: [Brain Infarction] explode all trees or MeSH descriptor: [Stroke] explode all trees or MeSH descriptor: [Brain Ischemia] explode all trees or MeSH descriptor: [Cerebral Infarction] explode all trees or MeSH descriptor: [Infarction, Middle Cerebral Artery] or "acute isch?emic stroke":ti,ab,kw or "acute cerebral isch?emia":ti,ab,kw or "brain isch?emia":ti,ab,kw) or ("brain ?edema":ti,ab,kw or "cerebral ?edema":ti,ab,kw or "midline shift":ti,ab,kw or MeSH descriptor: [Brain Edema])) and ((MeSH descriptor: [Ultrasonography, Doppler, Transcranial] explode all trees or "ultraso* Doppler transcranial":ti,ab,kw or "transcranial Doppler ultraso*":ti,ab,kw or "TCD":ti,ab,kw or "transcranial colo?r-coded sonography":ti,ab,kw or "TCCS":ti,ab,kw or "transcranial ultrasound":ti,ab,kw or "transcranial ultrasonography":ti,ab,kw or "transcranial sonography":ti,ab,kw) and (MeSH descriptor: [Tomography, X-Ray Computed] explode all trees or MeSH descriptor: [Neuroimaging] explode all trees or MeSH descriptor: [Magnetic Resonance Imaging] explode all trees or MeSH descriptor: [Cerebral Angiography] or MeSH descriptor: [Magnetic Resonance Angiography] explode all trees or "computed tomography":ti,ab,kw or "CT":ti,ab,kw "MRI":ti,ab,kw or "computed tomography angiography":ti,ab,kw or "CT angiography":ti,ab,kw)) |
