# Supplementary material for: Diagnostic value of transcranial ultrasonography for selecting subjects with large vessel occlusion: a systematic review
Source: Ultrasound J. 2019 Oct 22;11:29. doi: 10.1186/s13089-019-0143-6 (PMC6805840; doi:10.1186/s13089-019-0143-6)
Supplement: Supplementary file 2 — Additional file 2. Criteria for the diagnosis of arterial stenosis in individual studies. [file 13089_2019_143_MOESM2_ESM.docx]

**Additional file 2.** Criteria for the diagnosis of arterial stenosis in individual studies.

| **Reference** | **MCA** | **ICA** | **ACA** | **PCA** | **VA** | **BA** |
| --- | --- | --- | --- | --- | --- | --- |
| Akopov 2002 | Abnormal mean flow velocities if higher than the mean±2 SD of control population |  |  |  |  |  |
| Bar 2010 | MCA trunk ≥50% stenosis if PSV > 220 cm/s | Distal ICA ≥50% stenosis if PSV > 220 cm/s |  |  |  |  |
| Boddu 2011 | >50% stenosis – PSV 140 cm/s  >70% stenosis – PSV 180 cm/s | >50% stenosis – PSV 120 cm/s | | >50% stenosis – PSV 100 cm/s | | |
| Gerriets 2002 | ↑mean systolic blood flow velocity >120 cm/s;  a side-to-side difference in blood flow velocity >21%; spectral signs of disturbed flow |  |  |  |  |  |
| Guan 2013 | MV > 100 cm/s or SV > 160 cm/s |  | MV > 80 cm/s or SV > 120 cm/s | MV >70 cm/s or SV > 100 cm/s | | |
| Nasr 2013 | ≥50% stenosis threshold -  MCA (M1) 220 cm/s |  | ≥50% stenosis threshold –  ACA (A1) 155 cm/s | ≥50% stenosis threshold -  PCA (P1) 145 cm/s | ≥50% stenosis threshold -  120 cm/s | ≥50% stenosis threshold -  140 cm/s |
| Rathakrishnan 2008 | Moderate stenosis ≥ 80 cm/s | Chernyshev et al (2005), Demchuk et al (2000) |  | Chernyshev et al (2005), Demchuk et al (2000) | | |
| Seidel 2009 | max PSV >160 cm/s |  | |  | | |
| Tsivgoulis 2007 | Chernyshev et al (2005), Alexandrov et al (2004, 2007) | | MFV ≥80 cm/s and ≥30% difference compared with the contralateral ACA segment | Chernyshev et al (2005), Alexandrov et al (2004, 2007) | | |
| Tsivgoulis 2008 |  |  |  | 50%-stenosis MFV > 80 cm/s and when the stenotic-to-normal MFV ratio was 2 | | |
| Viola 1993 | Comparison was made with healthy controls | | | | | |
| Wada et al 2002 | M1 stenosis >50%: PSFV of M1 > 180 cm/s |  |  | PCA (P2 segment) stenosis > 50%: PSFV > 200 cm/s |  |  |
| Zubkov 2008 | Not specified | | | | | |

Abbreviations: ACA – anterior cerebral artery; AI – asymmetry index; BA – basilar artery; ICA – internal carotid artery; MCA – middle cerebral artery; MFV – mean flow velocity; PCA – posterior cerebral artery; PCom – posterior communicating artery; PSFV – peak systolic flow velocity; VA – vertebral artery.
