# Supplementary material for: Diagnostic value of transcranial ultrasonography for selecting subjects with large vessel occlusion: a systematic review
Source: Ultrasound J. 2019 Oct 22;11:29. doi: 10.1186/s13089-019-0143-6 (PMC6805840; doi:10.1186/s13089-019-0143-6)
Supplement: Supplementary file 3 — Additional file 3. Criteria for the diagnosis of arterial occlusion in individual studies. [file 13089_2019_143_MOESM3_ESM.docx]

**Additional file 3.** Criteria for the diagnosis of arterial occlusion in individual studies.

| **Reference** | **MCA** | **ICA** | **ACA** | **PCA** | **VA** | **BA** |
| --- | --- | --- | --- | --- | --- | --- |
| Akopov 2002 | Abnormal mean flow velocities if lower than the mean ± 2 SD of control population.  Distal MCA occlusion: decreased and low-resistance M1 MCA flow velocities with blunted MCA waveforms in distal MCA with signs of flow diversion to ipsilateral ACA. |  |  |  |  |  |
| Bar 2010 | MCA M1 occlusion: absent MCA flow signal + visualization of the remaining arteries of the anterior part of the circle of Willis.  Distal M1 segment occlusion or multiple MCA branch occlusions (M2): site difference of 30% in PSV values detected in the proximal part of the M1 segment | T-type ICA occlusion: M1, A1, and distal ICA segment flow signals were absent; simultaneously, ipsilateral or contralateral A1 visualization using the ipsilateral temporal bone window was necessary | Goertler, Allendoerfer and von Reutern (2002) |  | Goertler, Allendoerfer and von Reutern (2002) |  |
| Boddu 2011 | Not specified | | | | | |
| Brunser 2009 | Demchuk et al (2000), Alexandrov, Demchuk and Burgin (2002) | | | | | |
| Gerriets 2001 | Not specified | | | | | |
| Gerriets 2002 | MCA mainstem: no Doppler signal of the MCA with present flow in ACA and PCA |  |  |  |  |  |
| Goertler 1998 | Diminished flow velocity – PSV < 40 cm/s (as established in comparison to age-adjusted healthy subjects)  Interhemispheric asymmetry of MCA MFV– correlates well with multiple (>3) MCA branch occlusions, if extracranial ICA occlusions had been excluded.  Normal MCA PSV was set to 48 cm/s |  |  |  |  |  |
| Guan 2013 | Blunted signal - much lower than velocities of the ipsilateral ACA and PCA |  |  |  |  |  |
| Kadimi 2000 | Hyperechoic structure during real-time B- mode evaluation with no Doppler signal or waveform |  |  |  |  |  |
| Kenton 1997 | A negative AI = reduction in the MFV in the symptomatic side. An AI of -200% = occlusion of the main stem of the symptomatic MCA (flow velocity 0 cm/s). Threshold ±21%.  Occlusion = lack of signal on the colour display visualization of at least two other vessels and lack of any signal with PW Doppler |  |  |  |  |  |
| Nasr 2013 | MCA Main Stem Occlusion: absence of a colour Doppler flow signal and its Doppler spectrum in the location of the proximal MCA segment with sufficient visibility of the other arteries (ACA A1-segment, C1-segment of intracranial ICA) or veins (deep middle cerebral vein) of the anterior circulation or visibility of the contralateral anterior circulation.  The diagnosis of MCA branch occlusions is based on the calculation of the asymmetry index |  |  |  |  |  |
| Rathakrishnan 2008 | Demchuk et al (2000), Chernyshev et al (2005) | |  | Demchuk et al (2000), Chernyshev et al (2005) | | |
| Seidel 2009 | MCA (M1) occlusion: no MCA stem signal on the colour display, no Doppler signal when tracing the lateral fissure visible in B-mode, and detectable distal ICA or ACA Doppler signal.  MCA large branch or multiple branches (M2) occlusion: based on the AI: diminution of MFV of the MCA of 21% |  |  |  | | |
| Tsivgoulis 2007 | Proximal (M1) MCA occlusion: absence of flow or the presence of minimal, blunted, or damped flow signals throughout the MCA at an insonation depth of 45 to 65 mm, + flow diversion in the ipsilateral ACA or posterior cerebral artery.  Distal (M2) MCA occlusion: minimal, blunted, or damped flow signals at an insonation depth of 30 to 45 mm + the presence of flow diversion signals in ipsilateral neighbouring arteries (i.e., other  M2 branches) | TICA occlusion: absence of flow or the presence of minimal, blunted, or damped flow signals at an insonation depth of 60 to 70 mm accompanied by anterior cross-filing with flow reversal at the ipsilateral ACA or collateral flow with increased velocities in the ipsilateral-posterior communicating  artery | Absence of flow or the presence of minimal, blunted, or damped flow signals at an insonation depth of 62 to 75 mm | Demchuk et al (2000), Alexandrov et al (2004, 2007), Chernyshev et al (2005) | | |
| Tsivgoulis 2008 | MFV in the PCom was expected be equal or greater than the highest MFV velocity documented in the middle cerebral  artery | |  | Absence of flow (TIBI 0) or the presence of minimal (TIBI I), blunted (TIBI II), or dampened (TIBI III) flow signals. In case of proximal BA occlusion with distal flow reversal through PCom collateralization, the MFV in the PCom was expected to be equal or greater than the highest MFV velocity documented in the BA. Compensatory flow increase in the contralateral VA or cerebellar collaterals (arterial flow directed toward the probe with higher MFV than in VA at depths of 55 to 70 mm for the posterior inferior cerebellar artery, 75 to 85 mm for the anterior inferior cerebellar artery, and 90 mm for superior-cerebellar artery in the absence of PCom flow or suspicion of  the reversed BA flow signature) in case of terminal vertebral artery or BA occlusions | | |
| Viola 1993 | Comparison was made with healthy controls | | | | | |
| Wada 2002 | M1 occlusion (horizontal portion): no signal on the contralateral side of hemiparesis and MCA flow can be observed on the ipsilateral side of hemiparesis.  M1 occlusion of lower side: ED-ratio of M1 > 1.9 (side-to-side ratio of the end-diastolic flow velocity in M1 segment, which was calculated by dividing the velocity on the side of higher flow velocity by that on the contralateral side) |  |  | PCA (P2 segment) occlusion: no signal in P2 |  |  |
| Zubkov 2008 | Not specified | | | | | |

Abbreviations: ACA – anterior cerebral artery; AI – asymmetry index; BA – basilar artery; ICA – internal carotid artery; MCA – middle cerebral artery; MFV – mean flow velocity; PCA – posterior cerebral artery; PCom – posterior communicating artery; PSFV – peak systolic flow velocity; VA – vertebral artery.
