# Supplementary material for: Diagnostic value of transcranial ultrasonography for selecting subjects with large vessel occlusion: a systematic review
Source: Ultrasound J. 2019 Oct 22;11:29. doi: 10.1186/s13089-019-0143-6 (PMC6805840; doi:10.1186/s13089-019-0143-6)
Supplement: Supplementary file 4 — Additional file 4. The accuracy parameters of transcranial ultrasonography for detecting cerebral arterial stenosis or occlusion in individual studies. [file 13089_2019_143_MOESM4_ESM.docx]

**Additional file 4.** The accuracy parameters of transcranial ultrasonography for detecting cerebral arterial stenosis or occlusion in individual studies.

| **Reference** | **Stenosis/occlusion** | **Index test** | **Reference test** | **Se (%)** | **Sp (%)** | **PPV (%)** | **NPV (%)** | **Accuracy (%)** |
| --- | --- | --- | --- | --- | --- | --- | --- | --- |
| **Middle cerebral artery** | | | | | | | | |
| Bar 2010 | Occlusion | TCCS ± contrast | CTA | 92.3 | 94.4 | 92.3 | 94.4 | 93.5 |
| Boddu 2011 | >50% stenosis | TCD | MRA | 89 | 88 | 95.5 | 70.9 |  |
|  | >70% stenosis |  |  | 78 | 88.9 | 82 | 85 |  |
| Brunser 2009 | Occlusion | PMD-TCD | CTA | 95.6 | 96.2 |  |  | 96 |
| Guan 2013 | Stenosis or occlusion | TCD | CTA | 100 | 98.9 | 97.2 | 100 | 99.2 |
| Tsivgoulis 2007 | Occlusion | TCD | CTA | 90 | 97 | 90 | 97 |  |
| Wada 2002 | Occlusion/stenosis | TCCS | DSA | 100 | 98.9 |  |  | 98.9 |
| **Anterior cerebral artery** | | | | | | | | |
| Boddu 2011 | >50% stenosis | TCD | MRA | 87.7 | 98.7 | 86.7 | 96.6 |  |
| Guan 2013 | Stenosis or occlusion | TCD | CTA | 88.9 | 95.4 | 76.1 | 98.1 | 94.5 |
| **Terminal internal carotid artery** | | | | | | | | |
| Guan 2013 | Stenosis or occlusion | TCD | CTA | 90.9 | 98.2 | 83.3 | 99.1 | 97.6 |
| Tsivgoulis 2007 | Occlusion | TCD | CTA | 75 | 100 | 100 | 99 |  |
| **Internal carotid artery siphon** | | | | | | | | |
| Boddu 2011 | >50% stenosis | TCD | MRA | 90 | 97.9 | 85 | 96.4 |  |
| Guan 2013 | Stenosis or occlusion | TCD | CTA | 89.4 | 97.2 | 85 | 98.1 | 96 |
| **Posterior cerebral artery** | | | | | | | | |
| Boddu 2011 | >50% stenosis | TCD | MRA | 88 | 97.8 | 84.5 | 98.7 |  |
| Guan 2013 | Stenosis or occlusion | TCD | CTA | 81.8 | 95.2 | 78.2 | 96.1 | 92.9 |
| Tsivgoulis 2008 | Stenosis/occlusion | PMD-TCD | CTA, MRA, DSA | 80 | 99 | 66 | 99 | - |
| **Basilar artery** | | | | | | | | |
| Boddu 2011 | >50% stenosis | TCD | MRA | 92 | 96.6 | 83 | 99 |  |
| Guan 2013 | Stenosis or occlusion | TCD | CTA | 75 | 94 | 77.7 | 93.0 | 89.8 |
| Tsivgoulis 2007 | Occlusion | TCD | CTA | 33 | 99 | 50 | 98 |  |
| Tsivgoulis 2008 | Stenosis/occlusion | PMD-TCD | CTA, MRA, DSA | 75 | 99 | 75 | 99 |  |
| **Vertebral artery** | | | | | | | | |
| Boddu 2011 | >50% stenosis | TCD | MRA | 85 | 98 | 86.4 | 99 |  |
| Guan 2013 | Stenosis or occlusion | TCD | CTA | 63.4 | 96.5 | 89.6 | 84.8 | 85.9 |
| Tsivgoulis 2008 | Stenosis/occlusion | PMD-TCD | CTA, MRA, DSA | 66 | 98 | 72 | 98 | - |
| **Any cerebral artery** | | | | | | | | |
| Bar 2010 | Occlusion | TCCS ± contrast | CTA | 100 | 77.8 | 91.7 | 100 | 93.5 |
| Brunser 2009 | Occlusion | PMD-TCD | CTA | 81.8 | 94 |  |  | 90 |
| Rathakrishnan 2008 | Stenosis | TCD | CTA | 76.2 |  | 84.2 |  |  |
| Tsivgoulis 2007 | Occlusion | TCD | CTA | 79.1 | 94.3 | 87.2 | 90.3 | 89.4 |

Abbreviations: ACA – anterior cerebral artery; AI – asymmetry index; BA – basilar artery; CTA – computed tomography angiography; DSA – digital subtraction angiography; ICA – internal carotid artery; MCA – middle cerebral artery; MFV – mean flow velocity; MRA – magnetic resonance angiography; NPV – negative predictive value; PCA – posterior cerebral artery; PCom – posterior communicating artery; PMD – power motion-mode; PPV – positive predictive value; PSFV – peak systolic flow velocity; SD – standard deviation; Se – sensitivity; Sp – specificity; TCCS – transcranial colour-coded duplex sonography; TCD – transcranial Doppler sonography; VA – vertebral artery.
